# Voice-Based Screening for SARS-CoV-2 Exposure in Cardiovascular Clinics (VOICE-COVID-19-II): Protocol for a Randomized Controlled Trial

**DOI:** 10.2196/41209

**Published:** 2023-01-31

**Authors:** Emily Oulousian, Seok Hoon Chung, Elie Ganni, Amir Razaghizad, Guang Zhang, Robert Avram, Abhinav Sharma

**Affiliations:** 1 DREAM-CV Lab McGill University Health Centre McGill University Montreal, QC Canada; 2 Centre for Outcomes Research and Evaluation Research Institute of the McGill University Health Centre McGill University Montreal, QC Canada; 3 Division of Cardiology McGill University Health Centre McGill University Montreal, QC Canada; 4 Division of Cardiology University of Ottawa Ottawa, ON Canada; 5 Montreal Heart Institute University of Montreal Montreal, QC Canada

**Keywords:** voice-based technologies, Amazon, Alexa, SARS-CoV-2, COVID-19, digital screening

## Abstract

**Background:**

The COVID-19 pandemic has disrupted the health care system, limiting health care resources such as the availability of health care professionals, patient monitoring, contact tracing, and continuous surveillance. As a result of this significant burden, digital tools have become an important asset in increasing the efficiency of patient care delivery. Digital tools can help support health care institutions by tracking transmission of the virus, aiding in the screening process, and providing telemedicine support. However, digital health tools face challenges associated with barriers to accessibility, efficiency, and privacy-related ethical issues.

**Objective:**

This paper describes the study design of an open-label, noninterventional, crossover, randomized controlled trial aimed at assessing whether interactive voice response systems can screen for SARS-CoV-2 in patients as accurately as standard screening done by people. The study aims to assess the concordance and interrater reliability of symptom screening done by Amazon Alexa compared to manual screening done by research coordinators. The perceived level of comfort of patients when interacting with voice response systems and their personal experience will also be evaluated.

**Methods:**

A total of 52 patients visiting the heart failure clinic at the Royal Victoria Hospital of the McGill University Health Center, in Montreal, Quebec, will be recruited. Patients will be randomly assigned to first be screened for symptoms of SARS-CoV-2 either digitally, by Amazon Alexa, or manually, by the research coordinator. Participants will subsequently be crossed over and screened either digitally or manually. The clinical setup includes an Amazon Echo Show, a tablet, and an uninterrupted power supply mounted on a mobile cart. The primary end point will be the interrater reliability on the accuracy of randomized screening data performed by Amazon Alexa versus research coordinators. The secondary end point will be the perceived level of comfort and app engagement of patients as assessed using 5-point Likert scales and binary mode responses.

**Results:**

Data collection started in May 2021 and is expected to be completed in fall 2022. Data analysis is expected to be completed in early 2023.

**Conclusions:**

The use of voice-based assistants could improve the provision of health services and reduce the burden on health care personnel. Demonstrating a high interrater reliability between Amazon Alexa and health care coordinators may serve future digital tools to streamline the screening and delivery of care in the context of other conditions and clinical settings. The COVID-19 pandemic occurs during the first digital era using digital tools such as Amazon Alexa for disease screening, and it represents an opportunity to implement such technology in health care institutions in the long term.

**Trial Registration:**

ClinicalTrials.gov NCT04508972; https://clinicaltrials.gov/ct2/show/NCT04508972

**International Registered Report Identifier (IRRID):**

DERR1-10.2196/41209

## Introduction

The COVID-19 pandemic has led to over 551,000,000 cases and 6,300,000 deaths across the world [1]. This high rate of mortality can be partly attributed to an aging population and the fact that comorbidities such as cardiovascular disease significantly increase the risk of death. As a consequence of COVID-19’s infectiousness and high rate of mortality, the pandemic has exerted a significant pressure on health care resources such as the availability of health care professionals and their services [2]. As a result of this pressure, telemedicine and artificial intelligence (AI) technologies have proliferated to increase the efficiency of health care and address the unmet needs of a strained health care system [3]. Indeed, the rapid adoption of digital technology in health care delivery has increased as a result of the ongoing global digitalization [4]. For example, programs such as chatbots have gained popularity in promoting COVID-19 vaccination, and telemedicine-based health care delivery has become a patient-accepted and sustained addition to ambulatory care [5-7].

AI is a technology that enable machines to comprehend, act, and learn by simulating human-like intelligence. Machines and computer applications using AI can distinguish, classify, and retrieve information via algorithmic training [8]. These technologies are beneficial as they can be used in health care systems for intelligent screening, thus limiting contact between health care workers and patients. Additionally, intelligent screening systems such as interactive voice response systems have the potential to help control the spread of COVID-19 by streamlining patient monitoring, contact tracing, and continuous surveillance. However, a potential limitation of telemedicine and AI technologies is the potential for inaccurate data capture due to the absence of face-to-face communication. Telemedicine and AI technologies that use interactive voice response systems, such as Amazon Alexa, have been shown to be able to facilitate patient screening within a hospital environment [9]. In fact, previous research has demonstrated good agreement and high interrater reliability (IRR) between Amazon Alexa and health care providers [10]. However, such research has been primarily observational, resulting in a high potential for risk of bias from the priming effect, which can occur when an exposure to certain screening strategy influences responses to a subsequent one. In addition, it is unclear how such technologies perform when interacting with patients who are older and have multiple comorbidities such as those with heart failure (HF) [11]. Additional knowledge gaps include questions related to ethical and privacy concerns of patients exposed to digital tools [12].

To address this knowledge gap, we aim to conduct a two arm, crossover, randomized controlled trial to evaluate the accuracy of data capture by Amazon Alexa versus health care providers in the context of COVID-19 screening. In addition, patient-centric decision-making and concerns surrounding the use of such interactive voice response systems will be evaluated via Likert scales in a postscreening survey.

## Methods

### Overview

The study presents no more than minimal risk of harm to participants and involves no procedures for which written consent is required. All collected data will be anonymous, and all participants will be deidentified and given an identification number. No compensation will be offered to the participants.

### Study Design and Setting

The VOICE-COVID-19-II study is designed as an open-label, noninterventional, crossover, randomized controlled trial comparing the accuracy of data capture by Amazon Alexa versus health care providers in the context of SARS-CoV-2 screening. Patients visiting an HF clinic at the Royal Victoria Hospital (RVH) of the McGill University Health Center (MUHC), in Montreal, Quebec, will be eligible for inclusion. In total, 52 participants will be recruited at the RVH HF clinic. Patients will be randomized to first be screened for symptoms of SARS-CoV-2 by either Amazon Alexa or the research coordinator; participants will then subsequently cross over and be screened by either Amazon Alexa or the research coordinator. Each patient’s responses to both the Amazon Alexa– and research coordinator–administered survey will be compared.

**Figure 1:**
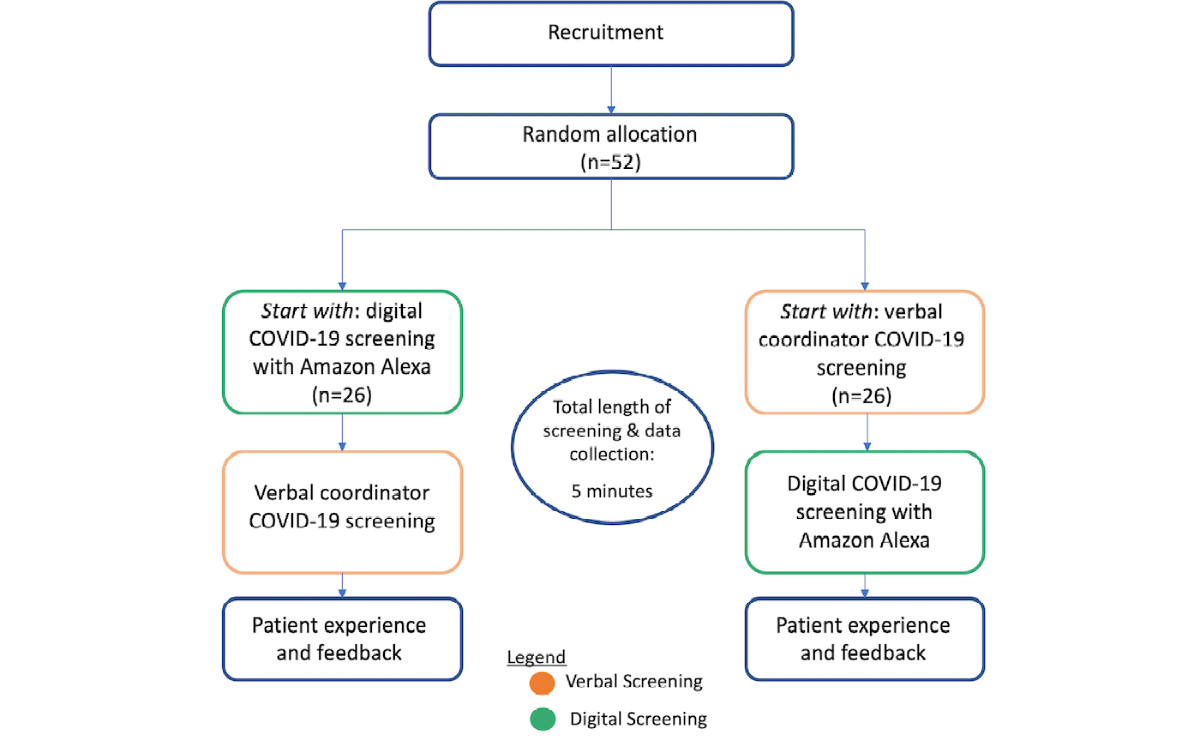
Consolidated Standards of Reporting Trials (CONSORT) diagram of the study.

The order of screening will be determined randomly by 1:1 block randomization. A flowchart of the study is shown in Figure 1. The Amazon device will not record any personal health information nor health data. After the completion of the SARS-CoV-2 screening, a survey will be presented by the coordinator to patients to assess their perceived user preference and Alexa software application engagement. The postscreening survey will evaluate the sense of privacy and data safety concerns regarding the use of digital screening, as well as the interest of patients in receiving voice response systems at home, experience with voice response systems, and interest for digital health care aid based on cohort demographics.

### Eligibility Criteria

Patients and employees aged older than 18 years at the RVH HF clinic will be eligible for inclusion. Individuals of all sexes, races, and ethnicities will be eligible to participate in the study. Exclusion criteria include inability to speak English or French and inability to interact with the device as defined by severe speaking impediments, hearing loss, and neurological problems.

### Ethics Approval

This study was approved by and received a waiver of documentation of consent from the institutional Research Ethics Board (REB; approval 2020-6583) at the MUHC. Any modification to the protocol will be reported to the REB (Multimedia Appendix 1).

### Device Used

The screening device that will be used is the Amazon Echo Show 8. With Amazon Web Services, an Alexa Skill will be developed using the Amazon Alexa Developer Kit. An Alexa Skill is an interactive voice interface that allows users to interact with a device, hands-free. Skills are based on vocal interaction models that help Alexa determine a user’s request. The voice algorithm is first based on automatic speech recognition (ASR), followed by natural language understanding (NLU). ASR is speech recognition software that converts spoken words in text. ASR helps detect audio waveform patterns, matching each waveform with sounds from a given language (English or French for this trial). However, because vocal intonations and inflections vary widely, NLU is used for the rearrangement of spoken data into a computer-readable format. NLU processes the request by decrypting the ASR-converted message. NLU interprets the input, extracts the data, and restructures it so that machines may understand and analyze it. Together, this allows for human-computer interaction, allowing devices to understand commands without the syntax of computer languages.

In this study, the Amazon Echo 8 will be used to screen patients for SARS-CoV-2 through a series of questions. The Alexa Skill built for this study is a predetermined sequence of screening questions triggered by a vocal request. Requests include answering a question by “yes” or ”no,” stating numbers (subject’s age and ID number), etc. To activate the SARS-CoV-2 screening process, a prespecified activating utterance is programmed: “Alexa, Clinic Visit.” Each response to a question act as a trigger for the next question. The questions are based on Health Canada recommendations and clinical consultations and will be regularly updated with new guidelines and approved vaccines. The skill will be available in both French and English. Therefore, patients will be able to choose their language of preference and interact with the device in the most effective way. The screening questions used for this study are available in Table 1. No personal health data will be recorded by the Amazon Echo 8.

Alexa will be used to collect and transfer data to secure servers owned by the principal investigator. The virtual servers are located in Canada and are protected behind a secure firewall. These servers undergo daily backups of the data in case of corruption. No personal health care information will be stored on these servers. Furthermore, a log will be kept of the IP addresses logging on the server.

**Table 1:** COVID-19 screening questions.

Screening questionnaire (English)	Response type
1. Welcome to check-in assist, *bienvenue dans* check-in assist. To continue in English, say English. *Pour continuer en français, dites français.*	Binary mode (English/French)
2. We can help you see if you have symptoms of the COVID-19 virus. What is your participant number?	Numbers
3. Do you work in the hospital?	Binary mode (Yes/No)
4. What is the reason for your visit? You can say appointment, visitor, delivery, pick-up or other.	Predetermined options
5. Have you traveled outside of Canada in the last 14 days?	Binary mode (Yes/No)
6. Have you been in close contact with a confirmed case of COVID-19 without protective equipment?	Binary mode (Yes/No)
7. Do you currently have any of the following symptoms: fever, runny nose, sore throat, or diarrhea?	Binary mode (Yes/No)

### Recruitment

Participant recruitment will be conducted in teams of 2 bilingual coordinators. A relative degree of quietude will be exercised to avoid issues of device communication during the digital screening portion of this study. Consequently, recruitment will be done in physicians’ offices when patients with HF are either waiting for their appointment or have completed their clinic visit. Patients will undergo the screening process in a designated office to ensure privacy.

During recruitment, the number of patients who accepted or refused to participate will also be recorded. At the beginning of every HF clinic, medical staff and physicians will be alerted of the presence of research coordinators on site. The team will obtain consent from the physicians on duty to collaborate with the patients in their offices. The Amazon Echo will then be plugged into the fully charged uninterrupted power supply (UPS) and connected to the Wi-Fi. Both the tablet and the Echo device will be configured, and the activation code will be spoken to verify if the Alexa Skill is still responding and functional.

Participants meeting the eligibility criteria will undergo SARS-CoV-2 screening by both Amazon Alexa and a research coordinator in random order. No personal health information or health data will be collected through the Alexa device. Verbal informed consent forms are approved by the REB. If the participant is eligible and informed consent has been obtained, past medical history data will be collected to analyze the study’s demographics. Participants will be informed that enrollment in the study is on a voluntary basis and that withdrawal from the study is possible at any stage. Withdrawal of consent will not affect subsequent medical treatment of patients nor their relationship with the treating physician.

To be able to move efficiently across the clinic, the clinical setup consisting of a tablet, the Amazon Echo, and a UPS on a mobile cart. A tablet will be used for Research Electronic Data Capture (REDCap) access, where randomization, coordinator screening, and patient feedback will be recorded. REDCap is a secure web application for building and managing online surveys and databases. Additionally, a UPS will be used to keep the Amazon Echo continuously powered. This will be done to avoid the reconfiguration period that occurs each time after the Amazon Echo device is powered off. The UPS used in this study will be a Schneider Electric 600 Volt-Amp.

Participants will remain 6 feet (1.83 m) away from the Amazon Echo. The 3 devices (Echo, tablet, and UPS) will be cleaned with bleach-based wipes in between every participant. The duration of the screening process is estimated to be 5 minutes.

### Data Collection

Patient recruitment and data collection will be conducted at the site of recruitment. Data related to the trial will be anonymized and recorded in a coded, password-protected digital file behind the coordinating center’s institution firewall. Only the principal investigator will have access to the code. All the information collected will remain confidential to the extent required and provided by law. The collected data will be kept for 25 years after the end of the study before being destroyed. There will be no data monitoring committee in this study as no therapeutic intervention is involved. Open access to the manuscript will not be provided.

### Randomization and Blinding

The order of screening is randomized as this study is a crossover trial with a higher potential for risk of bias from the priming effect. Participants will be randomly assigned to either start with the digital SARS-CoV-2 screening or with the coordinator screening. Randomization will be done in a 1:1 ratio using a randomization procedure generated by REDCap. Both participants and coordinators involved will not be blinded to the allocation process.

### Outcome Measures

The primary outcome of the study will be the concordance of screening for symptoms of SARS-CoV-2 carried out by the Amazon Alexa device compared with the manual screening carried out by a trained research coordinator. The secondary outcome will be a 5-point Likert scale and binary mode responses evaluating patients’ perceived comfort interacting with Alexa. The above outcomes will be measured as part of the postscreening survey carried out by the research coordinator at the end of the SARS-CoV-2 symptom-screening process and inputted into a secure web application form. The ease of the Amazon Alexa screening process, privacy and data safety concerns, interest in digital health care at home, and willingness to be vaccinated will be asked with a standard 5-point Likert scale. The relationship between the participant’s vaccine status and the answers regarding the participant’s comfort with digital health use, as well as the relationship between interest in voice response systems use at home and cohort demographics (eg, gender, age, and relevant medical history) will be determined by a linear regression model. Finally, any technical issues will be noted manually, on the REDCap platform, as well as any comments, remarks, and questions made by the research participants. These notes will be taken into consideration in the evaluation of the efficiency and accessibility of the digital Amazon screening. However, it is important to note that given that the survey questions are clear and objective, there is little risk of bias that in-person observations will affect the internal validity of the study’s results.

### Statistical Analysis

The coprimary end points for this study relate to the IRR on the accuracy of the randomized screening data from Amazon Alexa compared to coordinator data as the comparator. IRR will be evaluated using simple and weighted Cohen κ statistics (where possible) and percentage concordance between responses obtained from Amazon Alexa and research coordinators. The calculated κ scores will be compared between the 2 study arms for each screening question. In addition, linear mixed-effects model will be used to investigate differences in the 2 study arms, adjusting for the fixed effect of the screening order and the random effect of correlated observations in the demographic data. Furthermore, postscreening survey questions will be examined and presented on a Likert scale. This study will aim to recruit at least 52 participants, with minimally 26 participants in each study arm. The estimation of the sample size is based on the *kappaSize* package in R (version 4.1.2; R Foundation for Statistical Computing), with a 1-tailed test null κ score of 0.6 assuming a proportion of positive ratings of 0.9 and a preliminary κ score of 0.9 [13,14]. The sample size will also be adjusted with the power analysis of one-way ANOVA and the chi-square test to obtain a power of 0.8, accounting for a large effect size of above 0.4 and a significance level of .05. The demographic data will be presented in the baseline characteristics table, with categorical variables as counts (percentages) and continuous variables as medians and interquartile ranges.

### Intervention and Comparator

Participants allocated to the intervention group will initially undergo screening by the Amazon Alexa device for symptoms of SARS-CoV-2 (screening questions in Table 1). All questions, excluding the first question to determine the preferred language, allow for the collection of responses that serve as epidemiological or clinical risk factors that predict the risk of having an infection with SARS-CoV-2. These participants will then undergo the same screening process, but by a trained research coordinator. Meanwhile, patients allocated initially to the comparator group will first undergo screening by a trained research coordinator, followed by digital screening by the Amazon Alexa device.

At the end of this process, all participants will undergo a postscreening survey (Table 2) conducted by a trained research coordinator. The goal of this postscreening survey is to evaluate user preference, concerns about privacy and data storage, and the Alexa software application engagement. The survey will ask 11 questions about their screening experience and their perception of voice-based technology. Questions 1, 2, and 6 allow specific evaluation for participants comfort with utilizing technology. More specifically, answers to these questions could serve as markers for the device’s perceived functionality and reliability as well as possibility for remote screening. Questions 3, 4, and 5 allow the collection of responses to assess the patient’s perception relative to data security. Questions 7 and 8 measure the participant’s subjective perception of the device’s effectiveness. Questions 9, 10, and 11 assess the overall ease of use of the device.

Other variables will be collected in the postscreening phase of the study such as SARS-CoV-2 vaccine status, assigned sex at birth, and duration of digital screening. These variables will be utilized for stratification-based statistical analysis on the answers obtained in the postscreening survey.

Prespecified subgroups of interest include those by language (French and English), sex, and age.

**Table 2:** Postscreening survey questionnaire.

Postscreening survey questions	Response type
1. Did you feel comfortable speaking into the Amazon Alexa device?	5-point Likert scale
2. Have you used this type of device before or do you own this type of device?	Binary mode
3. Would you feel comfortable being screened for symptoms using the Amazon Alexa device?	Binary mode
4. Do you have any concerns about privacy or data safety?	5-point Likert scale
5. Do you feel your data would be kept secure by such a device?	5-point Likert scale
6. Can you see yourself using such a device at home to assist with any healthcare needs?	5-point Likert scale
7. Did you feel the check-in asked the screening questions appropriately?	5-point Likert scale
8. Did you feel the check-in minimized your chance of contracting COVID-19?	5-point Likert scale
9. Was the language easy to understand?	5-point Likert scale
10. Was the check-in easy to use?	5-point Likert scale
11. How would you rate this check-in overall?	5-point Likert scale

## Results

Funding for the VOICE-COVID-19-II trial was obtained in May 2021. The recruitment of participants for the VOICE-COVID-19-II trial started in May 2021, with simultaneous acquisition of data by the Amazon Echo Show 8 device, and is expected to be completed in fall 2022. Data analysis is expected to be completed in early 2023. Subsequently, trial results and analysis are expected to be published by mid-2023.

## Discussion

### Expected Findings

The study aims to test the concordance of screening for symptoms of SARS-CoV-2 done by Amazon Alexa compared to manual screening done by research coordinators. It also aims to evaluate the perceived comfort of patients when interacting with Alexa. Voice-based technology has the potential to improve clinical treatment for patients affected by SARS-CoV-2 by optimizing rapid and accurate data acquisition and can be extended to allow for screening of other conditions. Before implementing this interactive voice response system, the IRR of the program screening for the virus and overall patient experience, compared to the standard person-to-person screening, must be evaluated.

With standard methods of screening for symptoms and exposures, there is an increased burden on health care professionals that remains strenuous in the context of the pandemic. The use of commercially available digital tools to aid in screening for SARS-CoV-2 symptoms may be instrumental in reducing the burden on health care personnel, as well as minimizing opportunistic spread of disease via person-to-person contact [15].

A number of approaches using available digital health technologies to screen for SARS-CoV-2 symptoms are being explored, including automated text messaging and smartphone apps [16,17]. However, interactive voice response systems remain relatively unexplored. A previous study has evaluated the quality of health information dissemination on cancer screening provided by voice assistants, such as Siri, Alexa, and Cortana, but did not evaluate these devices’ ability to screen for cancer [18]. One study evaluated the quality of speech intelligibility of Amazon Alexa and Google Home by providing individuals with intellectual disabilities with smart speaker devices [19]. Another recent study screened patients with major depressive disorder with the Patient Health Questionnaire 9 via randomizing screening with Amazon Alexa compared to manual screening [20]. However, manual screening was done through paper format, and the time between digital and manual screening was 1 month. The potential ease of use of voice-based commands, which, when optimized, would better simulate standard person-to-person interactions compared to requiring digital input, could prove to be optimal in situations where the use of electronic recording of data may be difficult, such as with older participants. Furthermore, the use of Amazon Alexa, a voice response system available at home and other nonclinical environments, could provide additional advantages in terms of remote data collection. A reliable method of rapid acquisition of medical data easily accessible to patients could significantly facilitate future trials and personalizing health care delivery. Similar to other remote digital technology, such as Google Home or Apple’s Siri, voice response systems have potential for great scalability for larger participant numbers, given its commercial nature, cloud-based data collection, and easy access.

However, the increasing popularity of remote screening tools during the SARS-CoV-2 pandemic revealed a need for objective measures of the reliability of the data acquired by these tools. In particular, the previous phase of this study did not directly address the risk of bias in the virtual screening question answers when presented with the comparator set of in-person questions beforehand. The strengths of this study are founded upon its design to isolate a verified measure of the accuracy of the screening method and the use of commercial interactive voice response systems. This study aims to examine the reliability of the data acquired by the device while accounting for the priming bias inherent in previous similar studies, where participants would be affected by choices presented by the in-person set of screening questions used as the comparator. In addition, the Amazon Alexa program used in the skill set is based on a widely accessible commercial device designed for home use, which would provide a reasonable simulation of the technology that would be used for widespread public screening.

### Limitations

The study does have some limitations. Although the sample size may be adequate in detecting IRR between the virtual screening program and the standard in-person screening questionnaire, it is too small to detect the effectiveness of the questionnaire in triaging patients exposed to COVID-19. While the use a more extensive COVID-19 questionnaire may have increased the amount of data available for analysis, a shorter questionnaire developed with clinical experts was used to enhance patient recruitment. Given that the focus of this study was the accuracy of information obtained remotely compared to an in-person standard and not specifically to screen for SARS-CoV-2, insight into its capability as a screening measure was limited. However, regarding the diagnostic and screening accuracy of voice-based digital screening for COVID-19, a larger-scale study utilizing gold standard diagnostic assays (ie, reverse transcription polymerase chain reaction) would be required. Should a survey evaluating the accuracy of the questionnaire itself be proven to be effective, interactive voice response systems, such as Amazon Alexa, could provide users with a notification for their risk of a positive diagnosis and whether testing is encouraged given a posttest likelihood of illness.

In terms of the questionnaire, it is possible that some questions regarding personal contact with COVID-19 will have some interperson variability secondary to question interpretation. However, given the objective nature of the COVID-19 questionnaire as well as individuals’ lived experiences with the pandemic, the overall potential for risk of bias is low. Furthermore, although the study and questionnaire used are limited to recording answers in a 5-point Likert scale and a binary response of yes or no, speech AI is becoming increasingly sophisticated, with improved speech recognition and more flexible conversations in newly developing propriety speech AI. This could prove to be a unique strength of voice-based screening in future health care delivery, with a possibility of more personalized interactions with patients, nuanced screening questions, and more efficient information dissemination to patients in the form of an interactive question-and-answer program. As remote data collection develops over time, there will be new potential avenues of medical AI research for personalized health care delivery with intelligent voice-based assistants.

### Conclusion

In conclusion, the second phase of the VOICE-COVID-19-II randomized controlled trial aims to evaluate the accuracy of data capture by interactive voice response systems, such as Amazon Alexa, compared to health care providers as the baseline. If the study proves to be effective, it could open additional avenues of research and would provide a novel viewpoint on implementing digital health in the context of the pandemic, allowing for screening in both hospitals and at home.

## Appendix

Multimedia Appendix 1 Standard Protocol Items: Recommendations for Interventional Trials (SPIRIT) checklist.

## Abbreviations

AI: artificial intelligence
ASR: automatic speech recognition
HF: heart failure
IRR: interrater reliability
MUHC: McGill University Health Center
NLU: natural language understanding
REDCap: Research Electronic Data Capture
REB: Research Ethics Board
RVH: Royal Victoria Hospital
UPS: uninterrupted power supply

## Data Availability

In accordance with the approval granted for this study by the McGill Research Ethics Board, the data files will be stored securely and protected by Canadian virtual servers and a secure firewall. Patient level data will not be available. Request for the coding used in the Alexa Skill can be made available upon reasonable request to the corresponding author.
