# Authorship Correction: Voice-Based Screening for SARS-CoV-2 Exposure in Cardiovascular Clinics (VOICE-COVID-19-II): Protocol for a Randomized Controlled Trial

**DOI:** 10.2196/50893

**Published:** 2023-11-23

**Authors:** Emily Oulousian, Seok Hoon Chung, Elie Ganni, Amir Razaghizad, Guang Zhang, Robert Avram, Abhinav Sharma

**Affiliations:** 1 DREAM-CV Lab McGill University Health Centre McGill University Montreal, QC Canada; 2 Centre for Outcomes Research and Evaluation Research Institute of the McGill University Health Centre McGill University Montreal, QC Canada; 3 Division of Cardiology McGill University Health Centre McGill University Montreal, QC Canada; 4 Division of Cardiology University of Ottawa Ottawa, ON Canada; 5 Montreal Heart Institute University of Montreal Montreal, QC Canada

In “Voice-Based Screening for SARS-CoV-2 Exposure in Cardiovascular Clinics (VOICE-COVID-19-II): Protocol for a Randomized Controlled Trial” (JMIR Res Protoc 2023;12:e41209) the authors noted the following error:

In the author list, the first two authors Emily Oulousian and Seok Hoon Chung had contributed equally but this was not listed. In the corrected version the first two authors have been listed as:

Emily Oulousian*; Seok Hoon Chung*

*these authors contributed equally

The corrections will appear in the online version of the paper on the JMIR Publications website on November 23, 2023, together with the publication of this correction notice. Because this was made after submission to PubMed, PubMed Central, and other full-text repositories, the corrected article has also been resubmitted to those repositories.

